# Do retractile testes have anatomical anomalies?

**DOI:** 10.1590/S1677-5538.IBJU.2015.0538

**Published:** 2016

**Authors:** Kleber M. Anderson, Suelen F. Costa, Francisco J.B. Sampaio, Luciano A. Favorito

**Affiliations:** 1 Unidade de Pesquisa Urogenital, Universidade Estadual do Rio de Janeiro, RJ, Brasil

**Keywords:** Testis, Epididymis, Cryptorchidism, Retractile testicle, Anatomic

## Abstract

**Objectives:**

To assess the incidence of anatomical anomalies in patients with retractile testis.

**Materials and Methods:**

We studied prospectively 20 patients (28 testes) with truly retractile testis and compared them with 25 human fetuses (50 testes) with testis in scrotal position. We analyzed the relations among the testis, epididymis and patency of the processus vaginalis (PV). To analyze the relations between the testis and epididymis, we used a previous classification according to epididymis attachment to the testis and the presence of epididymis atresia. To analyze the structure of the PV, we considered two situations: obliteration of the PV and patency of the PV. We used the Chi-square test for contingency analysis of the populations under study (p <0.05).

**Results:**

The fetuses ranged in age from 26 to 35 weeks post-conception (WPC) and the 20 patients with retractile testis ranged in ages from 1 to 12 years (average of 5.8). Of the 50 fetal testes, we observed complete patency of the PV in 2 cases (4%) and epididymal anomalies (EAs) in 1 testis (2%). Of the 28 retractile testes, we observed patency of the PV in 6 cases (21.4%) and EA in 4 (14.28%). When we compared the incidence of EAs and PV patency we observed a significantly higher prevalence of these anomalies in retractile testes (p=0.0116).

**Conclusions:**

Retractile testis is not a normal variant with a significant risk of patent processus vaginalis and epididymal anomalies.

## INTRODUCTION

A retractile testis is defined as a supra-scrotal testis that can be manipulated easily into the scrotum and remain there without traction until the cremasteric reflex is induced (1). Recent studies generally urge observation of the evolution testicular position in cases of retractile testes (2, 3), because over 70% of patients with this condition show favorable evolution without the need for surgery (1). However, ascended testis or acquired undescended testis can occur in about 30% of cases (4).

Structural and ultrastructural studies have demonstrated morphological alterations in retractile testicle cases (5, 6), and one study of young adults who had been treated for retractile testis during the prepubertal period showed that only 28.5% had normal spermiograms (7).

Anomalies of the tunica vaginalis and the epididymis are associated with testicular torsion (8) and are very frequent in patients with cryptorchidism (9), but the anatomy of the processus vaginalis and mainly the relations between testis and epididymis in patients with retractile testis are unknown.

The objective of the present study was to assess the incidence of anatomical anomalies in patients with retractile testes.

## MATERIALS AND METHODS

This study was approved and was carried out in accordance with the ethical standards of the hospital's institutional committee on human experimentation.

We studied 62 patients prospectively with truly retractile testis during the period from January 2010 through January 2015. The retractile testis in this sample was defined based on physical examination findings. We included only patients with testis that can be brought down into the scrotum without tension and, after gentle massaging of the cord stay there upon release for a while.

We submitted to surgery 20 (32.25%) of the 62 patients. In eight cases of operated patients the retractile testis was bilateral. The surgery was performed because of parent anxiety and/or the impossibility for the periodic follow-up. We compared the anatomical findings of 28 retractile testes with 25 human fetuses (50 testes) with the testes in the scrotal position.

During the surgery, after the induction of anesthesia all patients had the testis in scrotal position and we used the trans-scrotal approach with a little midline scrotal incision with dissection of the cremaster muscle and fixation of the testis in dartos tunica in all cases.

The 25 fetuses were macroscopically well preserved. Their gestational age was determined in WPC, according to the foot-length criterion, which is currently considered the most acceptable parameter to calculate gestational age (10–12). The fetuses were also evaluated regarding crown-rump length (CRL) and body weight immediately before dissection. The same observer conducted the measurements.

After measurement, the fetuses were carefully dissected with the aid of a stereoscopic lens with 16/25X magnification. The abdomen and pelvis were opened to identify and expose the urogenital organs and inguinal canal and to show the testicular position. We observed patency of the processus vaginalis and the relationship between the testis and epididymis in fetuses and the patients.

To analyze the relations between the testis and epididymis in surgical patients and fetuses, we used a previous classification (13, 14): Type I - epididymis attached to the testis at the head and tail; Type II - epididymis totally attached to the testis; Type III - epididymis attached to the testis only at the head; Type IV - epididymis attached to the testis only at the tail; Type V - no visible connection between the testis and epididymis; and Type VI - epididymal atresia. Type I and II relationships are considered normal; while the other types are considered to be epididymal anomalies (EAs). To analyze the structure of the PV, we considered two situations: (a) complete obliteration of the PV between the internal inguinal ring and the upper pole of the testis; and (b) complete patency of the PV.

We used the Chi-square test for contingency analysis of the populations under study (p <0.05), calculated by the Graph Pad Prism software.

## RESULTS

The patients ranged in ages from 1 to 12 years old (average of 5.8). Table-1 reports the age of the patients, testicular position, PV patency and the presence of epididymal anomalies. The fetuses presented gestational ages between 25 to 35 WPC, weighed between 741 and 2600g, and had crown-rump length between 23 and 34cm. Of the 50 fetal testes, we observed complete patency of the PV in 2 cases (4%) and EAs in only 1 testis (2%). Table-2 reports the fetal parameters and the testis position. We observed two fetuses with patency of PV in the left testis and only one fetus had an epididymal anomaly (tail disjunction - Type III) on the right side.

**Table 1:** The table shows the age, the testicular position and the presence of patency of the processus vaginalis (PV) and epididymal anomalies. The patient number 10 had bilateral retractile testis with bilateral patency of processus vaginalis and epididymal anomaly in the left testis.

Patient	AGE	RT	LT	PV	Epididymis
1	1	Retractile	Retractile	Obliterated	Normal
2	2	Retractile	Scrotum	Obliterated	Normal
3	2	Scrotum	Retractile	Obliterated	Normal
4	3	Scrotum	Retractile	Obliterated	Normal
5	3	Scrotum	Retractile	Obliterated	Normal
6	3	Retractile	Scrotum	Patency (RT)	Anomaly in RT
7	3	Retractile	Scrotum	Obliterated	Normal
8	4	Scrotum	Retractile	Obliterated	Normal
9	5	Retractile	Scrotum	Obliterated	Normal
10	6	Retractile	Retractile	Patency Bil	Anomaly in LT
11	6	Retractile	Retractile	Obliterated	Normal
12	7	Retractile	Retractile	Obliterated	Normal
13	7	Retractile	Scrotum	Obliterated	Normal
14	8	Retractile	Retractile	Obliterated	Normal
15	8	Retractile	Retractile	Obliterated	Normal
16	9	Retractile	Retractile	Obliterated	Normal
17	9	Retractile	Scrotum	Patency (RT)	Anomaly in RT
18	11	Scrotum	Retractile	Patency (LT)	Normal
19	11	Retractile	Scrotum	Patency (RT)	Anomaly in RT
20	12	Retractile	Retractile	Obliterated	Normal

**Bil** = bilateral; **RT** = right testis and **LT** = left testis.

**Table 2:** The table shows the fetal age in weeks post conception (WPC) and the presence of epdidymal anomalies and patency of processus vaginalis (PV) in 25 fetus studied. The fetuses ranged in age between 25 to 35 WPC, weighted between 741 and 2600g, and had crown-rump length between 23 and 34 cm. The fetus number 2 and 3 had a PV patency in the left testis and only the fetus 10 had a epididymal anomaly (EA) a tail disjunction on the right side. RT = right testis and LT = left testis.

Fetus	Age (WPC)	RT	LT
1	25	Normal	Normal
2	26	Normal	**PV patente**
3	27	Normal	**PV patente**
4	27	Normal	Normal
5	27	Normal	Normal
6	27	Normal	Normal
7	28	Normal	Normal
8	28	Normal	Normal
9	28	Normal	Normal
10	28	**Tail disjunction**	Normal
11	28	Normal	Normal
12	28	Normal	Normal
13	28	Normal	Normal
14	28	Normal	Normal
15	28	Normal	Normal
16	29	Normal	Normal
17	29	Normal	Normal
18	30	Normal	Normal
19	30	Normal	Normal
20	31	Normal	Normal
21	31	Normal	Normal
22	32	Normal	Normal
23	33	Normal	Normal
24	35	Normal	Normal
25	35	Normal	Normal

**Figure 1:**
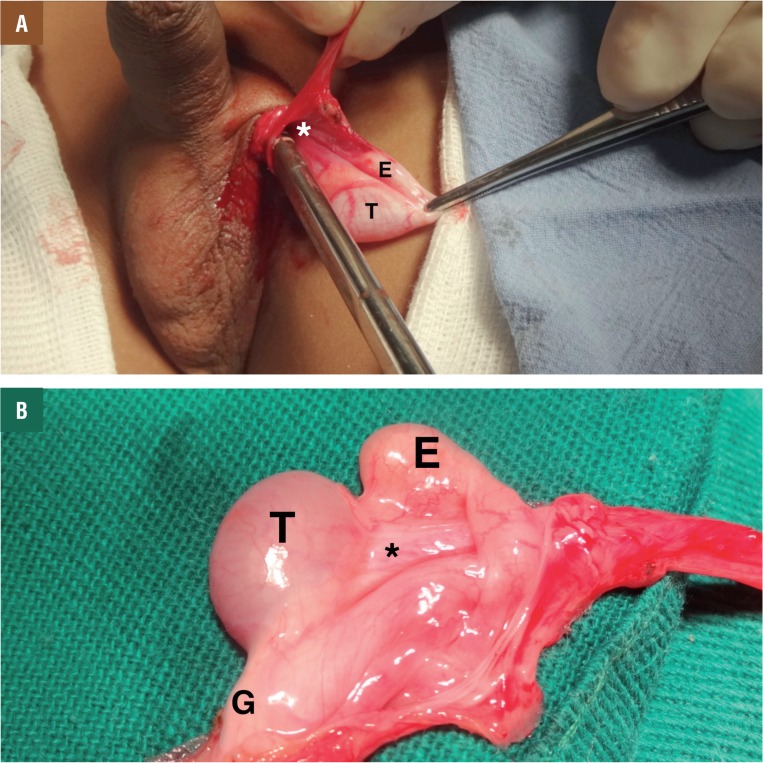
Anatomic anomalies in retractile testis. A) patient with 3 years-old with retractile testis presents complete patency of processus vaginalis (*). We can observe the surgical instrument inside the processus vaginalis. T=Testis and E=Epididymis. B) patient with 9 years-old with retractile testis presents the epididymis attached to the testis only at the head. T = Testis; E = Epididymis; G = gubernaculum and *Mesorquium.

Of the 28 retractile testes, we observed patency of the PV in 6 cases (21.4%) and EAs in 4 cases (14.28%). Of the 6 cases of PV patency, 4 (66.6%) were on the right side and 2 (33.3%) on the left side. Of the 4 cases of epididymal anomalies, 2 (50%) were on the right side and 2 (50%) on the left side. The majority of epididymal anomalies (3 - 75%) were tail disjunction (Type III – Figure-1) and only in one case (25%) did we observe total disjunction of the epididymis (Type IV). One of the patients had bilateral retractile testes with bilateral processus vaginalis patency and epididymal anomaly in the left testis.

When we compared the incidence of EAs and PV patency in the retractile testes with the fetuses, we observed a significantly higher prevalence of these anomalies in retractile testes (p=0.0116).

## DISCUSSION

Retractile testis has traditionally been considered a variant of normal testis because it usually descends into the scrotum during adolescence (15). In general, patients with retractile testis are periodically reviewed until the end of adolescence or until the testis has completely descended into the scrotum. According to the guidelines of the European Association of Urology, cases of retractile testis do not warrant medication or surgical intervention, and instead should only be monitored periodically until adolescence (16). Nevertheless, this condition can cause discomfort and also worry parents, sometimes prompting the choice for surgery to bring the affected testis into the scrotum.

Although the question is controversial, some authors have reported histological changes and spermiogram abnormalities in follow-up of adult patients with retractile testis (7, 17). Previous studies suggest surgical correction is necessary in some cases to prevent histological alterations in the germinative epithelium of patients with retractile testis (6, 18). Some previous studies conducted with boys with retractile testis reported that 18 to 32% of patients required surgical correction due to the development of ascending testes or decreases in testicular volume (1, 4), although in a retrospective study with 274 retractile testis only 6.9% of the patients needed surgical intervention; showing that the incidence of ascending testis is not always as high as has been reported in other studies (19).

In an interesting study with 3433 boys the authors observed that the prevalence of undescended testis in 6-year, 9-year and 13-year olds had a variation from 1.2% to 2.2% and after the age of 5 years, only acquired UDT was observed (20).

Studies applying ultrasound confirm that retractile testes show reduced volume in relation to normal testes (21). One recent retrospective study of 43 boys who had been diagnosed as having retractile testis noted that surgical intervention had been found necessary in 16.3% of the cases and the probability of surgery was higher in cases that had been diagnosed at younger ages (22).

Agarwal (4), in an important study, analyzed 204 retractile testicles and observed a risk of ascending testis in more than 30% of the cases. Of the patients in this study, surgery was performed on 61 testes and in 13% of the cases the processus vaginalis was found to be patent, while in the other cases of surgical intervention there was only observation of fibrous vestige of the processus vaginalis (4). The authors concluded that retractile testis can not be considered a normal variant because of the high risk of ascension and patency of the processus vaginalis.

In our sample, in which surgery was performed on 28 retractile testes, we found processus vaginalis patency in 21.4% of the cases. These findings confirm that the chance of patients with retractile testis presenting patent processus vaginalis is not negligible. In the control group composed of fetuses in which the testes had completed their migration, patency was only observed in 4% of the cases, a much lower rate than in the patients with retractile testis.

Cryptorchidism can be associated with various anatomical anomalies, but epididymal anomalies and patency of the processus vaginalis are among the most frequent. Epididymal anomalies are associated with cryptorchidism in over one-third of these cases (23, 24). Another study showed that individuals without cryptorchidism have a very low incidence of epididymal anomalies (13). Furthermore, human fetuses without apparent anomalies present epididymal anomalies in less than 3% of the cases, regardless of the testicular position (14). Epididymal anomalies can be classified as disjunction or atresia (13) and can be associated with infertility.

Patients with disjunction anomalies (head, tail or total disjunction) can present a longer distance between the testis and epididymis, the region called the mesorchium (8, 13, 14). Testicular torsion can be intravaginal or extravaginal. Intravaginal testicular torsion can occur because of an anomaly in the implantation of the tunica vaginalis (bell-clapper deformity) or due to the presence of an elongated mesorchium because of disjunction anomalies of the epididymis (8). Therefore, patients suffering from epididymal anomalies face a higher risk of developing intravaginal testicular torsion (8).

The rate of epididymal anomalies in patients with retractile testis is not well defined in the literature. In our sample, we observed that 14% of the patients with retractile testis submitted to orchiopexy presented epididymal anomalies. In three cases we observed tail disjunction, an anomaly where the mesorchium is elongated, and in one case there was total disjunction between the testis and epididymis, a situation associated with infertility and also increased size of the mesorchium.

This article presents the first description in the literature of the presence of epididymal anomalies associated with retractile testes. Despite the small sample, these findings can be significant. Future studies with larger samples will be necessary to confirm this association between epididymal anomalies and retractile testes, to provide further evidence that retractile testis is not a normal variant and does need treatment.

The main limitation of this work is the small sample of patients with retractile testis, but because of the controversy over treatment, surveys with large samples of patients having this condition who underwent surgery are not common in the literature. Another limitation is the control group. The ideal control group would be boys without inguinal-scrotal anomalies having the same average age as the group with such anomalies. However, ethical considerations regarding use of living subjects and the extreme rarity of cadavers to study requires the use of human fetuses with testicles located in the scrotum as the control group.

## CONCLUSIONS

Retractile testis is not a normal variant with a significant risk of patent processus vaginalis and epididymal anomalies.
